# Transitions Between Self and Proxy Completion of Nursing Home Resident Care and Activity Preferences

**DOI:** 10.1093/geroni/igaf122.3040

**Published:** 2025-12-31

**Authors:** Laura Block, Tonya Roberts

**Affiliations:** University of Wisconsin-Madison, Madison, Wisconsin, United States; University of Wisconsin-Madison, Madison, Wisconsin, United States

## Abstract

Identification of nursing home (NH) resident preferences for care and activities is crucial to informing person-centered care. Preferences are systematically recorded in the Minimum Data Set (MDS) and assessed through self and proxy report. Concerns surrounding accuracy of proxy report exist. Yet, transitions between self and proxy report of preferences, related predictors, and impact on preference patterns are unknown. This retrospective cohort study identified all residents with two preference assessments completed in the 2019 MDS 3.0 (N = 357,321) and examined transitions across preference respondent. Of the residents for whom preference respondent changed (n = 40,799), 31.8% transitioned from self to family report while 14% transitioned from self to staff report. In contrast, 21.8% transitioned from family to resident report and 12% from staff to resident report. Rates of dementia were only somewhat higher among residents who transitioned from resident to family (50.0%) versus family to resident report (46.5%). Cognition worsened in all cases of loss of self-report and improved in all cases of resuming self-report. Preferences reported as important varied across reporter. Findings have implications for understanding the potential impact of proxy report of preference reports and the importance of early preference assessment among individuals with dementia. Future research examining a wider range of factors that predict return of self-report capacity is needed.

